# Biocompatible Gold Nanoparticles Ameliorate Retinoic Acid-Induced Cell Death and Induce Differentiation in F9 Teratocarcinoma Stem Cells

**DOI:** 10.3390/nano8060396

**Published:** 2018-06-01

**Authors:** Sangiliyandi Gurunathan, Jin-Hoi Kim

**Affiliations:** Department of Stem Cell and Regenerative Biotechnology, Konkuk University, Seoul 05029, Korea

**Keywords:** teratocarcinoma stem cells, gold nanoparticles, luteolin, oxidative stress, differentiation, apoptosis

## Abstract

The unique properties of gold nanoparticles (AuNPs) have attracted much interest for a range of applications, including biomedical applications in the cosmetic industry. The current study assessed the anti-oxidative effect of AuNPs against retinoic acid (RA)-induced loss of cell viability; cell proliferation; expression of oxidative and anti-oxidative stress markers, pro- and anti-apoptotic genes, and differentiation markers; and mitochondrial dysfunction in F9 teratocarcinoma stem cells. AuNPs were prepared by reduction of gold salts using luteolin as a reducing and stabilizing agent. The prepared AuNPs were spherical in shape with an average diameter of 18 nm. F9 cells exposed to various concentrations of these AuNPs were not harmed, whereas cells exposed to RA exhibited a dose-dependent change in cell viability and cell proliferation. The RA-mediated toxicity was associated with increased leakage of lactate dehydrogenase, reactive oxygen species, increased levels of malondialdehyde and nitric oxide, loss of mitochondrial membrane potential, and a reduced level of ATP. Finally, RA increased the level of pro-apoptotic gene expression and decreased the expression of anti-apoptotic genes. Interestingly, the toxic effect of RA appeared to be decreased in cells treated with RA in the presence of AuNPs, which was coincident with the increased levels of anti-oxidant markers including thioredoxin, glutathione peroxidases, glutathione, glutathione disulfide, catalase, and superoxide dismutase. Concomitantly, AuNPs ameliorated the apoptotic response by decreasing the mRNA expression of *p53*, *p21*, *Bax*, *Bak*, *caspase-3*, *caspase-9*, and increasing the expressions of *Bcl-2* and *Bcl-Xl*. Interestingly, AuNPs not only ameliorated oxidative stress but also induced differentiation in F9 cells by increasing the expression of differentiation markers including *retinoic acid binding protein*, *laminin 1*, *collagen type IV*, and *Gata 6* and decreasing the expressions of markers of stem cell pluripotency including *Nanog*, *Rex1*, *octamer-binding transcription factor 4*, and *Sox-2*. These consistent cellular and biochemical data suggest that AuNPs could ameliorate RA-induced cell death and facilitate F9 cell differentiation. AuNPs could be suitable therapeutic agents for the treatment of oxidative stress-related diseases such as atherosclerosis, cancer, diabetes, rheumatoid arthritis, and neurodegenerative diseases.

## 1. Introduction

The therapeutic use of gold dates back millennia in Chinese, Arabian, and Indian societies. The recent development of nanoscience and nanotechnology has spurred the use of gold nanoparticles (AuNPs) in diagnostics, therapy, prevention, and hygiene. These uses reflect the unique properties that include physical, chemical, and optical behaviors; high surface reactivity; biocompatibility; resistance to oxidation; plasmon resonance (PR); and lack of toxicity [1,2]. AuNPs are basically non-toxic, thermally stable, can be easily synthesized, and have the potential for surface functionalization. These attributes make AuNPs a suitable platform for biomedical applications.

Previous studies indicated the potential of gold compounds as anti-inflammatory agents through their ability to inhibit the expression of nuclear factor-kappa B and subsequent inflammatory reactions, and their potential anti-oxidative effect in the treatment of ischemia and cerebral damage in rats and diabetic mice [1,3,4]. Furthermore, AuNPs exhibited potential therapeutic behavior against pathological neovascularization, rheumatoid arthritis, and neoplastic disorders by inhibiting vascular permeability factor/vascular and endothelial growth factor 165-induced proliferation of endothelial cells [5]. Recently, we reported that the anti-oxidative property of biologically synthesized AuNPs ameliorates cold and heat stress-induced oxidative stress in *Escherichia coli* [6]. A considerable amount of evidence suggests that AuNPs can promote cell osteogenic differentiation and mineralization. For instance, gelatin-chitosan composite capped AuNPs can be an efficient matrix for the growth of hydroxyapatite crystals [7]. AuNPs also reportedly facilitate the differentiation of bone marrow-derived mesenchymal stem cells (MSCs) to osteoblasts instead of adipocytes by the activation of the p38 mitogen-activated protein kinase signaling pathway [8]. Interestingly, AuNPs promote osteogenesis of adipose-derived MSCs through Wnt/β-catenin and osteogenic differentiation of osteoblasts [9,10,11]. Gold nanowires and nanoparticle-embedded biomimetic scaffolds promote the assembly of cardiac cells into elongated and aligned tissues [12,13]. Recently, these multi components composite could inhibits apoptosis of PC12 cells and dopaminergic neurons in Parkinson's disease (PD) models both in vitro and in vivo [14]; indicating significant potential therapeutic effects of AuNPs for PD.

Retinoic acid (RA) is a developmental morphogen that regulates cell division and differentiation in development by modulating *HOX* gene expression, and also determines spatial body axis orientation during embryogenesis [15]. RA is a potent and widely-used signaling cue that stimulates oxidative stress and differentiation of embryonic stem cells (ESCs) and stem/progenitor cells in vitro [16,17]. RA is frequently used as a differentiation agent in a variety of cells including SH-SY5Y [18], skeletal myoblasts, and neuroblasts [19]. In addition, the role of RA as an anticancer agent has been assessed in lung cancer [20], skin cancer [21], cutaneous T-cell lymphoma [22], and acute promyelocytic leukemia [23]. RA-induced differentiation therapy is a potential approach for the treatment of acute promyelocytic leukemia (APL) and to prevent cancer [24]. Several studies have provided evidence that the agonistic or antagonistic activity of retinoid analogs could inhibit growth and induce apoptosis in cancer cells [25]. RA-induced cell death with characteristic features of apoptosis has been demonstrated in a variety of cell lines including HeLa and HL-60 [26]. All-trans RA (ATRA) modulates the expression of many DNA damage response (DDR) proteins, including ataxia-telangiectasia mutated (ATM), tumor protein 53 (TP53), B-cell lymphoma 2 (Bcl-2), and caspases, suggesting that ATRA can modulate DDR [27,28]. Tokarz et al. [29] observed that ATRA increases the level of intracellular reactive oxygen species (ROS) and oxidative stress-induced DNA damage in ARPE-19 cells.

Although RA induces differentiation in a variety of cell lines, it induces oxidative stress, which is a major mediator of apoptosis. Accordingly, in several systems, oxidative stress-induced apoptosis can be inhibited by antioxidants and enzymes involved in the catabolism of ROS such as superoxide dismutase (SOD) and catalase (CAT) [30]. Paradoxically, higher concentrations of RA and its prolonged use can potentially induce apoptosis, rather than cell differentiation, in a variety of cell lines including F9 cells. In advanced or recurrent malignant diseases, RA is not very effective even at doses that are toxic to the host. Insight into the molecular mechanisms that regulate differentiation and inhibit RA-induced apoptosis in teratocarcinoma stem cells, and identification of agents that protect or restore the ability of cells to undergo differentiation may be crucial for more effective differentiation-mediated cancer therapies. Development of novel biocompatible agents and combination regimens are required. AuNPs may be an alternate differentiation agent that simultaneously overcomes apoptosis during differentiation and induces differentiation in a non-toxic manner. This potential is hampered by the lack of knowledge about the complex molecular mechanisms involved in the protective effect of AuNPs in RA-induced apoptotic cell death.

The effect of AuNPs on the differentiation of F9 ESCs was chosen as the differentiation model. F9 mouse embryonic teratocarcinoma cells have been used as a model for the analysis of molecular mechanisms associated with differentiation. In addition, this cell line is able to differentiate into visceral endoderm when treated with RA. The effects of AuNPs on RA-induced apoptosis in mouse embryonal teratocarcinoma cells have not been studied in detail.

Presently, AuNPs were synthesized using luteolin and were then characterized. The relative use of the luteolin as capping agents provides stability to gold nanoparticles. The protection afforded by these NPs to RA-mediated, oxidative stress-induced damage to F9 cells was investigated and the mechanism clarified. Finally, we investigated the potential role of AuNPs on the differentiation of F9 cells.

## 2. Results and Discussion

### 2.1. Synthesis and Characterization of AuNPs Using Luteolin

**Figure 1:**
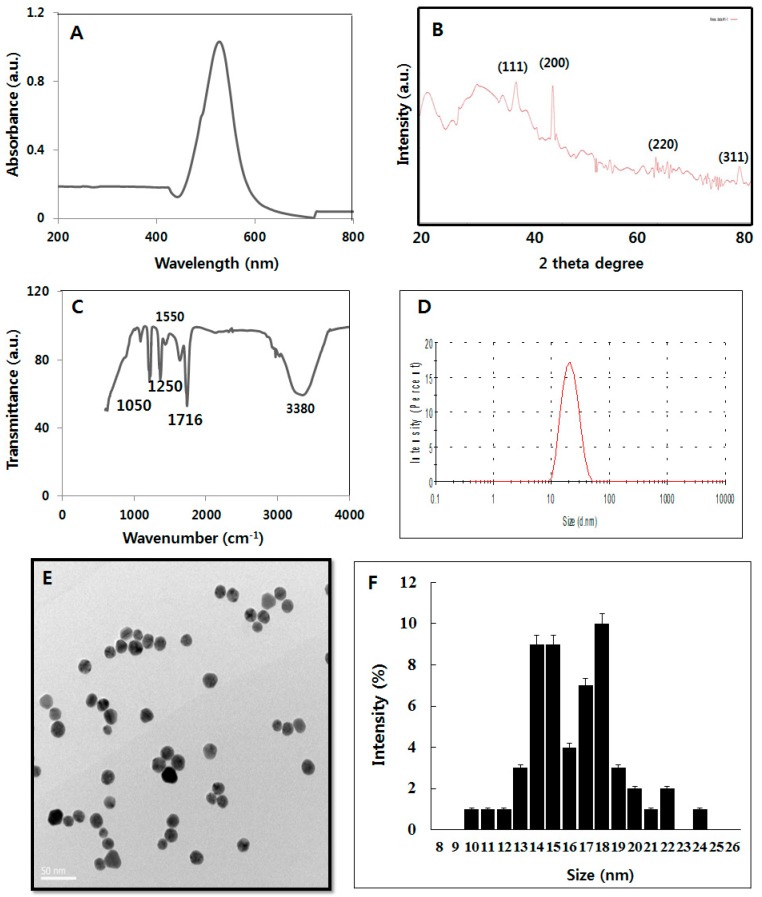
Synthesis and characterization of AuNPs using luteolin. Synthesis of AuNPs was performed by incubating luteolin (20 μM) and 1 mM aqueous HAuCl_4_ at 40 °C for 2 h. (**A**) Ultraviolet-visible spectroscopy of AuNPs revealed a maximum absorption peak at approximately 530 nm. This band was assigned to surface plasmon resonance of the particles; (**B**) XRD images of AuNPs; (**C**) Fourier transform infrared images of AuNPs; (**D**) Dynamic light-scattering (DLS) spectra of dispersions of AuNPs; (**E**) TEM images showing the size and shape of AuNPs; (**F**) Particle size distribution from TEM images. At least 200 particles were measured for each sample to obtain the size distribution. The average diameter was 18 nm. At least three independent experiments were performed for each sample and reproducible results were obtained. The data present the results of a representative experiment.

Generally, AuNPs exhibit localized surface PR (LSPR) between 500 nm and 600 nm [31,32]. Ultraviolet-visible spectroscopy, which is a valuable tool and simple method to assess the formation of AuNPs, was used to determine the peak of absorbance of AuNPs prepared by luteolin as a reducing and stabilizing agent. Biomolecule-assisted AuNPs display a peak at 530 nm, which is the typical surface PR (SPR) band of AuNPs (Figure 1A). Flavonoids are a diverse group of polyphenolic compounds that have important medicinal properties and act as reducing agents for the synthesis of NPs. Similarly, Levchenko et al. [33] synthesized biocompatible AuNPs using bioflavonoids such as rutin, quercetin, and luteolin as reducing agents and stabilizers using different ratios of HAuCl_4_ and luteolin.

X-ray diffraction (XRD) analysis was performed to determine the crystalline nature of the synthesized particles. The XRD pattern of AuNPs exhibited four different prominent Bragg reflections at approximately 38.4°, 44.8°, 65.0°, and 78.0° corresponding to different respective crystal planes of (111), (200), (220), and (311) (Figure 1B). The XRD facets of the biomolecule-mediated synthesis of AuNPs strongly agreed with the data of the Au standard published by the Joint Committee on Powder Diffraction Standards (file No. 04-0784). The mean size of the AuNPs was calculated using the Debye-Scherer equation by determining the width of the (111) and the similar Bragg reflection [33,34]. The synthesized AuNPs displayed an average size of 18 nm, which matched the particle size obtained from transmission electron microscopy (TEM). The XRD pattern clearly showed that the AuNPs formed by the reduction of AuCl_4_ions by luteolin are crystalline in nature. The pattern strongly corresponded to the crystalline planes of the face-centered-cubic structured Au.

Functional groups responsible for the reduction of auric chloride (AuCl_3_) by luteolin were determined by Fourier transform infrared spectroscopy (FTIR). As shown in Figure 1C, a wide variety of functional groups were present in the synthesized AuNPs, including carbonyl compounds (1716 cm^−1^), aromatic rings (1550 cm^−1^), amines (1250 cm^−1^), and alcohols (3380 and 1070 cm^−1^). These groups are common promoter agents in flavones for the bio-reduction of Au NPs, such as –OH and –COOH. In addition, the band observed at 1720 cm^−1^ could be assigned to the vibrational modes of C=C double bonds of these molecules. The large peak between 1250 and 1716 cm^−1^ fell in the region of carbonyl (C=O) stretching frequency and the bands at 3380 cm^−1^ corresponded to carbonyl and hydroxyl functional groups in alcohols. Thus, FTIR analysis allowed the identification of C=O that facilitated the reduction process and helped stabilize the generation of NPs.

Size distribution analysis was done using dynamic light scattering (DLS) and NP morphology was determined using TEM. DLS analysis revealed that the prepared AuNPs had an average size of 18 nm (Figure 1D), which exactly matched the size measured by TEM. TEM analysis also revealed the significantly uniform size and spherical shape of the luteolin-capped AuNPs (Figure 1E). A histogram plot of the size distribution estimated from TEM images revealed a range from 10–24 nm (average 18 nm; Figure 1F). The size distribution was narrow (±1.5 nm). TEM images also revealed the relatively high monodispersity compared to a prior description of chemically-mediated synthesis of AuNPs using tris(hydroxymethyl) aminomethane [35]. The analyses of the AuNPs prepared using luteolin strongly agreed with AuNPs formed using various biologic systems, including cellular extract of *Bacillus licheniformis* [6], *Brevibacterium casei* [33], mycelial extract of *Ganoderma* spp. [36], *B. flexus* [37], and *B. clausii* [38].

### 2.2. Dose-Dependent Effect of AuNPs on Cell Viability and Cell Proliferation of F9 Cells

**Figure 2:**
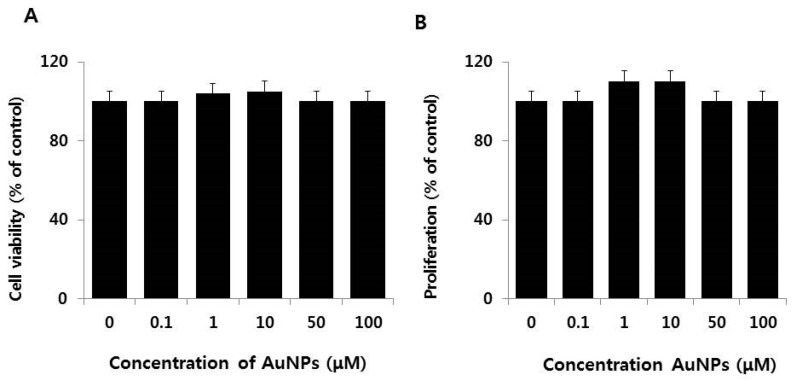
Effect of AuNPs on cell viability and proliferation of F9 cells. The cell viability (**A**) and proliferation; (**B**) of F9 cells were determined after a 24 h exposure to different concentrations of AuNPs (0.1–100 μM). At least three independent experiments were performed for each sample. The treated groups showed no statistically significant differences from the control group by the Student’s *t*-test.

Before investigating the ability of AuNPs to protect against RA and the embryonic differentiation of F9 cells, it was crucial to demonstrate that AuNPs did not adversely affect F9 cell viability and proliferation. As shown in Figure 2A, cell viability was not compromised by treatment with AuNPs at concentrations of up to 100 μM. Interestingly, exposure of F9 cells to a lower concentration (1–10 μM) of AuNPs promoted cell proliferation. Cell proliferation was not impeded by AuNPs concentrations of up to 100 μM (Figure 2B). Similarly, Pan et al. [39] reported cytotoxicity of various particles sizes in connective tissue fibroblasts, epithelial cells, macrophages, and melanoma cells. The toxicity was observed with a lower size of 1–2 nm compared to 15 nm. AuNPs with an average size of approximately 15 nm are non-toxic at up to 100-fold higher concentrations. Recently, Hau et al. [40] observed that AuNPs 10 nm in diameter were non-toxic to LOVO cells. Consistent with our findings, Connor et al. [41] reported that citrated and biotinylated 18 nm diameter AuNPs applied at lower concentrations did not induce toxicity in K562 leukemia cells. Chueh et al. [42] screened for the cytotoxic effects of AuNPs in different mammalian cell lines using various AuNPs concentrations ranging from 36 to 1000 ng/mL, and observed a concentration-dependent decrease in cell growth. The inhibitory growth effect was associated with the induction of apoptosis in Vero cells, but not in MRC-5 or NIH3T3 cells. Koch et al. [43] demonstrated that AuNPs were not cytotoxic and did not induce apoptotic cell death in N9 murine microglia and SH-SY5Y human neuroblastoma cells. Liu et al. [44] reported that AuNPs with a diameter of 20 nm promoted the proliferation of MC3T3-E1 cells in time- and dose-dependent manners. Collectively, the data indicate the biocompatibility of AuNPs.

**Figure 3:**
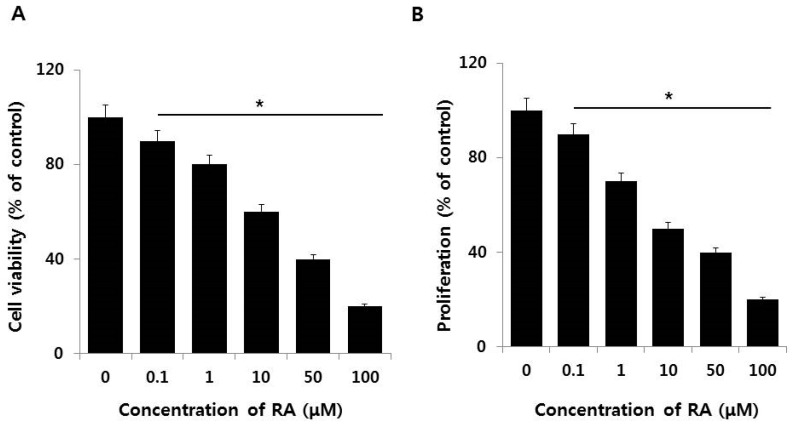
Effect of RA on cell viability and proliferation of F9 cells. The viability (**A**) and proliferation (**B**) of F9 cells were determined after a 24 h exposure to different concentrations of AuNPs (0.1–100 μM). At least three independent experiments were performed for each sample. The treated groups showed statistically significant differences from the control group by the Student’s *t*-test (* *p*, 0.05).

The dose-dependent effect of RA on F9 cell viability and proliferation inhibits growth and causes apoptosis in neuroblastoma cell lines [45,46,47]. To determine the potential effect of RA on F9 cell viability and proliferation, exponentially growing F9 cultures were treated with various concentrations of RA for 24 h. RA significantly and dose-dependently lessened the viability and proliferation of F9 cells (Figure 3A,B). The growth and proliferation of SK-PN-DW human primitive neuroectodermal tumor cells and SK-N-MC human neuroblastoma tumor cells is also impeded by RA (84% and 92%, respectively) in comparison with an untreated control [48]. An RA concentration of 10 μM produced a 50% inhibition of cell viability and cell proliferation, and was selected for further experiments.

### 2.3. Effect of AuNPs on RA-Induced Cell Death and Proliferation

**Figure 4:**
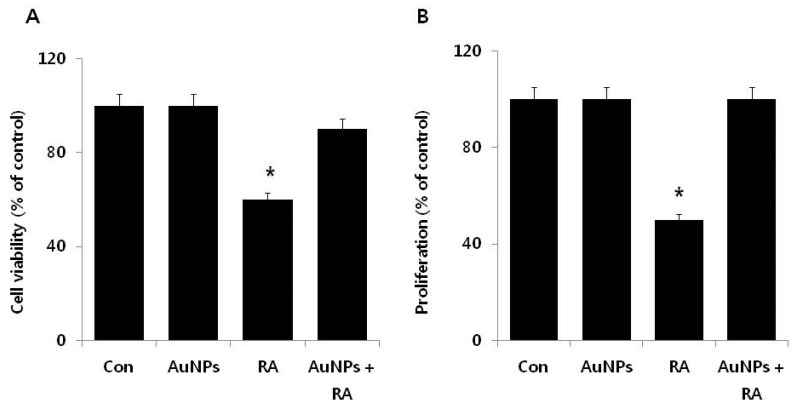
Effects of AuNPs on RA-induced cell death in F9 cells. The cell viability (**A**) and proliferation (**B**) of F9 cells were determined after a 24 h exposure to AuNPs (10 μM), RA (10 μM), and RA in the presence of AuNPs (both 10 μM). At least three independent experiments were performed for each sample. The treated groups showed statistically significant differences from the control group by the Student’s *t*-test (* *p*, 0.05).

To explore if AuNPs had a protective effect on the RA-induced cell death in F9 cells, cell viability and proliferation were determined using CCK-8 and BrdU assays. As expected from the preceding experiments, F9 cells treated with 10 μM of the AuNPs were unaltered in their viability and proliferation, whereas cells treated with 10 μM RA displayed significant reductions in cell viability and proliferation. When F9 cells were treated with 10 μM RA in the presence of 10 μM AuNPs, the RA-induced cell death was recovered by approximately 30–40% compared to the control, which indicated a protective effect of the AuNPs (Figure 4A,B). When compared to cell viability, the rescue effect of AuNPs was more pronounced (approximately 50%) for cell proliferation compared to the control. These findings support the potential proliferation efficiency of AuNPs. Recently, Xiao et al. [49] found that AuNPs modified with 6-mercaptopurine (6 MP) and a neuron-penetrating peptide (RDP) increased the proliferation and neurite growth of SH-SY5Y human neuroblastoma cells by increasing cellular metabolic activity compared to the control cells, which was due to the very efficient penetration of the 18 nm AuNPs into the cells. Gunduz et al. [50] demonstrated that intracellular accumulation of AuNPs leads to the inhibition of macropinocytosis and ultimately reduces endoplasmic reticulum stress. The results suggest that F9 cells treated with RA in the presence of AuNPs display increased cell viability and proliferation as compared to cells treated solely with RA. As found before, AuNPs did not adversely affect cell viability and cell proliferation.

### 2.4. Effect of AuNPs on RA-Induced Cytotoxicity

**Figure 5:**
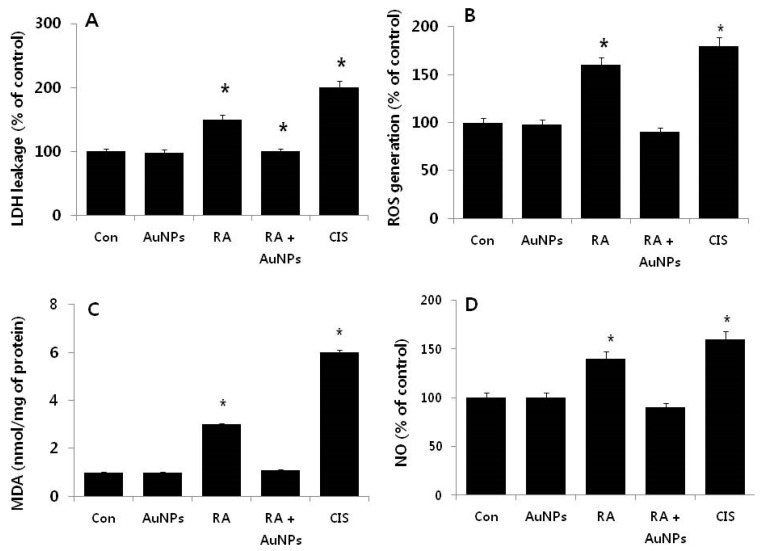
Effects of AuNPs on RA-induced cytotoxicity in F9 cells. F9 cells were treated with AuNPs (10 μM), RA (10 μM), RA and AuNPs (both 10 μM) and CIS for 24 h and the LDH activity (**A**) or ROS production (**B**) was measured at 490 nm using the LDH cytotoxicity kit. Relative fluorescence of 2′,7′-dichlorofluorescein was measured at an excitation wavelength of 485 nm and emission wavelength of 530 nm using a spectrofluorometer. F9 cells were treated with AuNPs (10 μM), RA (10 μM), and RA in the presence of AuNPs (both 10 μM) for 24 h, and the concentration of MDA (**C**) and NO (**D**) was measured and expressed as nanomoles/mg protein. At least three independent experiments were performed for each sample. The results are expressed as the mean ± standard deviation of three independent experiments. The treated groups showed statistically significant differences from the control group by the Student’s *t*-test (* *p*, 0.05).

To demonstrate the consistency of the loss of cell viability and cell proliferation caused by RA, we tested whether AuNPs were protected against RA-induced cytotoxicity in F9 cells. Cytotoxicity was determined by monitoring the leakage of lactate dehydrogenase (LDH), generation of malondialdehyde (MDA), and the generation of nitric oxide (NO). To test the rescue effect of AuNPs on RA-induced toxicity, F9 cells were exposed to 10 μM AuNPs, 10 μM RA, and 10 μM of each, and the LDH assay was performed. In order to compare the treated cells we used cisplatin (CA) as a positive control. After treatment with AuNPs, there is no leakage of LDH, indicating the absence of toxicity. By contrast, cells exposed to RA for 24 h displayed significant leakage of LDH, indicating disruption of the cell membrane. There was no significant difference among the groups treated with AuNPs and the control. In another study, rat liver cells exposed to 10 μM RA also displayed increased leakage of LDH and decreased cell viability [51]. Presently, in cells treated with RA in the presence of AuNPs, the leakage of LDH was not significant and was comparable with the untreated control. This indicated that the potential membrane disruption due to RA was prevented by the AuNPs (Figure 5A).

ROS is critical for oxidative stress. Oxidative stress is responsible for cell death and can regulate various signaling pathways involved in the differentiation of hematopoietic lineages, macrophages, or neuroblastoma cell lines [52,53]. Thus, we next examined the intracellular level of ROS to determine the involvement of oxidative stress in RA-induced oxidative stress (Figure 5B). Cells were treated with AuNPs (10 μM), RA (10 μM), or both (10 μM each) for 24 h. After treatment with AuNPs, no increase in the level of ROS compared to the control was evident, whereas cells treated with RA for 24 h displayed a significant amount of ROS level, indicating RA-induced cytotoxicity. There was no significant effect on ROS production when cells were treated with RA in the presence of AuNPs (Figure 5B). When compared to the control group, cells treated for 24 h with AuNPs prior to RA exposure did not display a significant level of ROS production, indicating that AuNPs could modulate RA-induced toxicity caused by the generation of ROS.

To detect the effect of RA on the cellular redox status in F9 cells, antioxidant defense system capability was assessed by examining lipid peroxidation. Lipid peroxidation refers to the oxidation of lipids by free radicals. It is one of the main manifestations of oxidative damage in tissues and cells [54]. Presently, we monitored the levels of thiobarbituric acid reactive substances (TBARS) levels as an indicator of lipid peroxidation. TBARS levels increased in F9 cells treated for 24 h with 10 μM RA compared to the control groups (Figure 5C). Surprisingly, the level of MDA was not significantly increased in cells treated with AuNPs or cells treated with RA in the presence of AuNPs.

Nitric oxide (NO) regulates multiple processes in cellular systems including neuronal development, plasticity, and differentiation and is a mediator of neurotoxicity in neuroblastoma cells [55]. NO synthases (NOSs) are a family of enzymes involved in NGF-induced differentiation of PC12 cells. NOS can induce growth arrest, neuronal differentiation, and neuritogenesis by modulating various signaling pathways [56]. Since NO is an important mediator for oxidative stress-induced neuronal damage, we determined if AuNPs could inhibit RA-induced NO production, When F9 cells were exposed to RA (10 μM) for 24 h, the production of NO was increased compared to the control. However, cells pretreated with AuNPs or cells treated with RA in the presence of AuNPs displayed significantly diminished production of NO to levels almost identical to those in the control cells (Figure 5D). Some brain cholinergic neurons can express neuronal NOS (nNOS), which results in free radical production that has been implicated with some forms of neurodegeneration. For example, treatment of SN56 cells with 1 μM RA for 48 h substantially increased nNOS mRNA (198%). The cells became vulnerable to excess NO and exhibited increased nuclear DNA fragmentation [57]. In TGW-nu-I neuroblastoma cells exposed to various concentrations of RA from 100 pM to 5 μM, RA treatment induced nNOS protein expression within 24 h in a concentration-dependent manner. The highest nNOS expression was induced by 5 μM RA [55]. Several studies suggest that vitamin A supplementation can increase mitochondrial superoxide anion production and induce lipid peroxidation, protein carbonylation, protein nitration, and oxidation of protein thiol groups in mitochondrial membranes isolated from a rat’s cerebral cortex, cerebellum, substantia nigra, striatum, frontal cortex, and hypothalamus [58]. Interestingly, our findings suggest that treatment with AuNPs, which largely inhibited the RA-mediated increase in NO production, can eventually reduce free radical production and oxidative stress in F9 cells.

### 2.5. Effect of AuNPs on RA-Induced Mitochondrial Dysfunction

**Figure 6:**
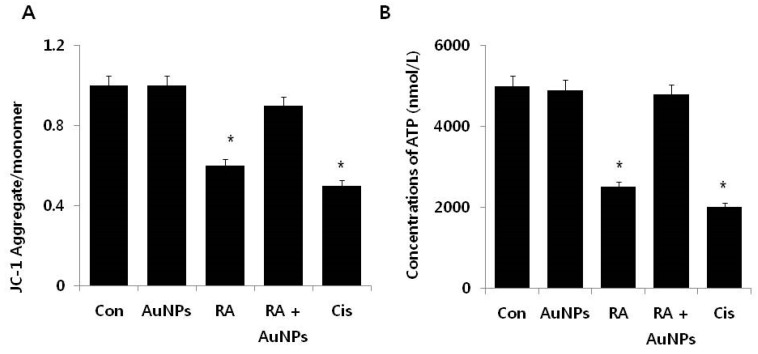
Effects of AuNPs on RA-induced mitochondrial dysfunction in F9 cells. (**A**) F9 cells were treated with AuNPs (10 μM), RA (10 μM), and RA in the presence of AuNPs (both 10 μM) for 24 h and the MMP was determined using the cationic fluorescent indicator JC-1; (**B**) ATP level was measured in F9 cells exposed to AuNPs (10 μM), RA (10 μM), and RA in the presence of AuNPs (both 10 μM).

Mitochondrial dysfunction and oxidative stress are primary factors for a variety of diseases. This dysfunction has been observed in the early stages of apoptosis [59]. Mitochondrial permeability transition (MPT) refers to the regulated opening of a large, nonspecific pore in the inner mitochondrial membrane [59]. MPT causes the loss of the mitochondrial membrane potential (MMP) [60]. To determine the role of the protective effect of AuNPs on RA-induced loss of MMP in F9 cells, the cells were treated with AuNPs (10 μM), RA (10 μM), and with RA in the presence of AuNPs for 24 h. After treatment with AuNPs, no significant difference was observed compared to the control. By contrast, cells treated with RA for 24 h displayed a significant loss of MMP, implicating RA as a cause of mitochondrial dysfunction in F9 cells (Figure 6A). When compared to the control group, cells treated with RA in the presence of AuNPs displayed no significant effect regarding the loss of MMP, indicating that AuNPs are able to protect cells from a loss of MMP in the presence of RA. In this experiment, cisplatin was used as the control. Cells treated with cisplatin displayed significant loss of MMP after 24 h of incubation, indicating that cisplatin could modulate toxicity via the loss of MMP. Our results agree with the recent demonstration that mitochondria pre-incubated with RA accumulates Ca^2+^ and inhibits the depolarization of MMP [61]. The authors described that increasing concentrations of RA impaired mitochondrial dysfunction in a manner that was directly proportional to RA concentration, suggesting that high concentrations of RA permeabilize the membrane to protons, possibly due to proton leakage through the Fo fraction of complex V. RA-induced hepatotoxicity due to the induction of MPT and alterations in bio-energetic parameters; the combination of RA with the anti-estrogen, endoxifen (EDX), reduced mitochondrial dysfunction [61]. Collectively, the findings suggest that mitochondrial function is an important factor for apoptosis and substantiated the potential of AuNPs as a suitable and alternative biocompatible agent to reduce apoptosis in stem cells.

Mitochondrial dysfunction is directly related to decreased complex I-III, complex II, succinate dehydrogenase (SDH), complex II-III, and complex IV enzyme activity, and also to decreased rates of ATP production and the increased rate of free radical formation [62]. Therefore, we were interested in determining whether mitochondrial dysfunction such as the loss of MMP is related to the decreased level of ATP production. The level of ATP was determined in cells treated with AuNPs (10 μM), RA (10 μM), andRA in the presence of AuNPs (both 10 μM) for 24 h. After treatment with AuNPs no significant difference was observed in the ATP level between the control and the AuNP treated group, whereas the ATP level was decreased by 50% in cells treated with RA for 24 h. Cells treated with RA in the presence of AuNPs displayed no significant loss of ATP, indicating the potential of AuNPs to abrogate RA-mediated mitochondrial dysfunction. In the control, cisplatin dramatically reduced the level of ATP production (Figure 6B). Elsewhere, apoptosis was induced in Sertoli cells exposed to retinol by a mitochondria-dependent pathway that ultimately decreased cell viability and ATP content and increased free radical formation [63]. The authors also reported that retinol increased the release of cytochrome c to the cytosol and consequently increased caspase-3 and caspase-7 [63]. Acitretin at concentrations ranging from 5 to μM was reported to alter the function of rat liver mitochondria by impairing phosphorylation capacity, with decreased ATP levels and adenine nucleotide translocase content, and Ca^2+^-induced mPTP [64]. ARPE-19 cells exposed to 10–30 μM all-*trans*-retinal displayed decreased viability and induced activation of oxidative stress-dependent Bax via phospholipase C/inositol triphosphate/Ca^2+^ signals and by activation of p53 following DNA damage [65].

### 2.6. Effect of AuNPs on RA-Induced Expression of Anti-Oxidative Stress Markers

**Figure 7:**
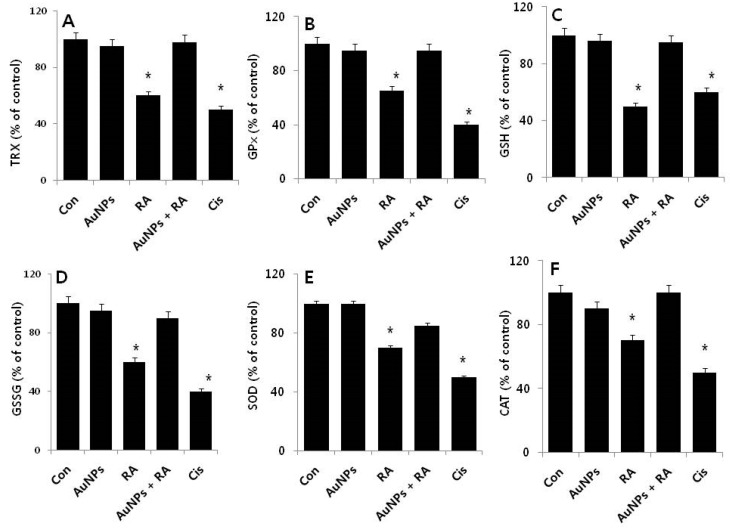
Effect of AuNPs and RA on the expression of anti-oxidative stress markers in F9 cells. F9 cells were treated with AuNPs (10 μM), RA (10 μM), and RA in the presence of AuNPs (both 10 μM) for 24 h. After incubation, the cells were harvested and washed twice with ice-cold PBS solution. The cells were collected and disrupted by ultrasonication for 5 min on ice. (**A**) The concentration of TRX was measured as nanomole/mg protein; (**B**) The specific activity of GPx measured as unit per mg protein; (**C**) GSH was measured as mg/g protein; (**D**) The ratio GSSG was measured as mg/g protein; (**E**) The specific activity of SOD was measured as unit/mg protein; (**F**) The specific activity of CAT was expressed as unit/mg protein. The results are expressed as mean ± standard deviation of three independent experiments. There was a significant difference in the treated cells compared to that of the untreated cells by the Student’s *t*-test (* *p*, 0.05).

ROS generated in response to endogenous and exogenous stimuli is an important factor for various cellular processes including growth, migration or differentiation, and apoptosis. However, excess ROS production induces apoptosis due to oxidative stress [66,67]. Increased levels of ROS leads to tumor initiation and progression and also a higher level of oxidative stress, which increases the vulnerability of the already damaged cells [68]. Therefore, manipulating ROS levels by redox modulation becomes an effective therapeutic approach to selectively kill cancer cells without causing significant toxicity to normal cells [69,70]. Based on this background, we are interested in examining the effect of AuNPs on RA-induced various anti-oxidative stress markers in F9 cells. The markers we selected were thioredoxin (TRx), glutathione peroxidases (GPx), glutathione (GSH), glutathione disulfide (GSSG), CAT, and superoxide dismutase (SOD). To determine the cellular level of these anti-oxidants, cells were treated with AuNPs (10 μM), RA (10 μM), and RA in the presence of AuNPs (both 10 μM) for 24 h. Treatment with AuNPs produced no significant difference between the control and treated cells. However, in cells treated with RA for 24 h, all the anti-oxidants were significantly reduced. Surprisingly, in cells treated with RA in the presence of AuNPs, no significant effect on the loss of anti-oxidants was observed, indicating that AuNPs were protective (Figure 7). Similarly, it was reported that AuNPs can control GSH, SOD, catalase, and GPx in diabetic mice to normal levels, by inhibiting the formation of ROS, lipid peroxidation, and scavenging of free radicals [1].

The accumulation of cellular ROS is mainly regulated by a series of enzymatic and non-enzymatic redundant endogenous antioxidant defense systems, which either prevent or scavenge ROS [71]. Antioxidant enzymes such as SOD, CAT, and GPx are responsible for the removal of free radicals and also act in concert with other proteins, such as TRX) and low-molecular-weight antioxidants including GSH, SOD, GSSG, CAT, and GPx to eradicate ROS and restore the reduced protein and lipid pools [72]. The possible mechanism of the protective effect of AuNPs against RA-induced oxidative stress could be the increased level of ROS and NOS. The decreased level of anti-oxidant proteins could be a later event of oxidative stress-mediated apoptosis manifested primarily through modifications of outer membrane proteins and lipids, causing the release of pro-apoptotic mitochondrial proteins, which initiate caspase-dependent and caspase-independent forms of cell death [43]. Collectively, our data provide significant evidence to substantiate the ability of AuNPs to alter physiological functions within F9 cells.

### 2.7. Effect of AuNPs and RA on Expression of Pro- and Anti-Apoptotic Genes in F9 Cells

**Figure 8:**
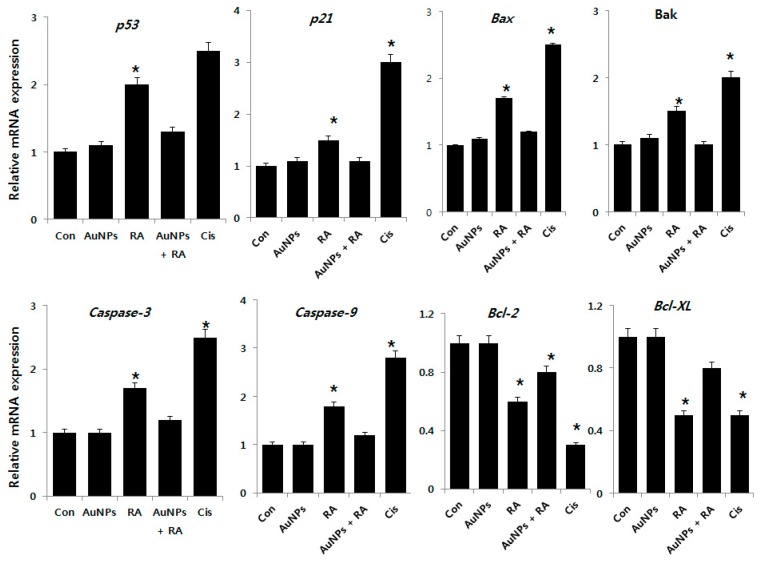
Effect of AuNPs on RA-induced expression of apoptotic gene expression in F9 cells. The expression of *p53*, *p21*, *Bax*, *Bak*, *caspase-3*, *caspase-9*, *Bcl-2*, and *Bcl-Xl* genes was measured in F9 cells exposed to AuNPs (10 μM), RA (10 μM), and RA in the presence of AuNPs (both 10 μM) for 24 h. After 24 h treatment, the fold-level of expression was determined in reference to expression values of GAPDH. Results are expressed as fold-changes. At least three independent experiments were performed for each sample. The treated groups showed statistically significant differences from the control group by the Student’s *t*-test (* *p*, 0.05).

To investigate the molecular mechanism of the effects of AuNPs on RA-induced apoptosis, we evaluated the expression of *p53*, *p21*, *Bax*, *Bak*, *caspase-3*, *caspase-9*, *Bcl-2*, and *Bcl-Xl*, which are involved in apoptosis as key regulators. Analysis by real-time PCR revealed that RA treatment strongly increased the mRNA expression of all but *Bcl-2* and *Bcl-Xl* from 1–3-fold after RA treatment (Figure 8). In contrast, *Bcl-2* and *Bcl-Xl* expressions were markedly down-regulated. Similarly, in keratinocytes exposed to ATRA, the mRNA expression of *p53* and *caspase-3*, *-6*, *-7*, and *-9* were reported to be markedly increased [27]. Previous findings also confirmed that the intrinsic pathway is engaged by cellular stress induced by RA by the involvement of Bcl-2 proteins that promote (Bax/Bak) or inhibit (Bcl-2/Bcl-xl) apoptosis through mitochondrial outer membrane permeabilization (MOMP) [73]. One of the key genes downstream of the DNA damage checkpoint is the tumor suppressor gene p53. Activated p53 in turn activates the target genes involved in growth arrest, DNA repair, and apoptosis. p53 has extra-nuclear apoptotic functions. It can bind the anti-apoptotic Bcl-2 proteins (Bcl-2 and Bcl-x) and can activate the pro-apoptotic multidomain proteins (Bax and Bak) to induce cytochrome C release and subsequent apoptosis [74]. ATRA inhibits cell migration, cell-cycle procession, invasiveness and proliferation, and promotes apoptosis [75]. Interestingly, cells treated with AuNPs did not display significant differences in their expression of pro- and anti-apoptotic genes at the tested concentrations. Bao et al. [76] reported that p21 is directly activated by RA in lymphoma cells and that the upregulation of p21 accompanies caspase-3 activation and precedes the occurrence of apoptosis. Several other studies found that retinoid-induced apoptosis via the expression of caspases in normal human epidermal keratinocytes, human leukemia cells, spontaneously immortalized human keratinocytes (HaCaT) cells, and ovarian carcinoma cells [77,78,79]. RA-induced pro-apoptotic genes including *tumor necrosis factor-related apoptosis-inducing ligand (TRAIL)* and *Apo2L/TNFSF10*, and anti-apoptotic genes including *cellular inhibitor of apoptosis protein-2 (cIAP2)* in human breast cancer cells [80]. AuNPs inhibits RA-induced upregulated expression of pro-apoptotic genes and downregulated expression of anti-apoptotic genes. Thus, AuNPs are implicated as a potential candidate for the prevention of apoptosis induced by RA.

### 2.8. Differentiation Effect of AuNPs and RA in F9 Cells

**Figure 9:**
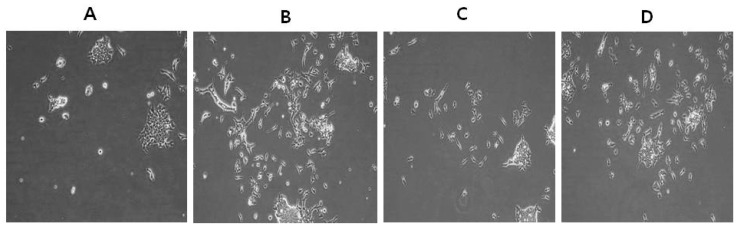
Effects of AuNPs and RA treatment on the differentiation of F9 cells. AuNPs-induced differentiation of F9 cells was determined after 24 h exposure to AuNPs (10 μM), RA (10 μM), and RA in the presence of AuNPs (both 10 μM). Phase contrast microscopy images showing the morphological changes in F9 cells after treatment with AuNPs and RA in 1% serum-supplemented medium. At least three independent experiments were performed for each sample. Control (**A**); AuNPs (10 μM) (**B**); RA (10 μM) (**C**); AuNPs and RA (**D**). (The magnification is 100 micro meter).

We next sought to demonstrate the potential ability of AuNPs to protect oxidative stress induced by RA and to assess the ability of RA to induce differentiation in F9 cells. We first observed the extension of differentiation, a typical neuronal phenotype after a 24 h exposure of AuNPs or RA (Figure 9). F9 teratocarcinoma stem cells usually grow in culture as closely-packed colonies; it is difficult to distinguish cell-cell boundaries in control cells (Figure 9A). F9 cells cultured in the presence of AuNPs for 24 h showed typical characteristic features of differentiation and resembled the F9 cell differentiated shape (Figure 9B), whereas RA-treated cells exhibited differentiated shapes (Figure 9C). Although RA induces differentiation effectively in a variety of cell lines including teratocarcinoma stem cells, ESCs, keratinocytes, and SH-SY5Y neuroblastoma cells, RA also potently induces cell death. The latter hinders the use of RA as a differentiation agent [16,27,81,82]. Interestingly, cells treated with RA in the presence of AuNPs exhibited reduced cell death and increased prevalence of the differentiation phenotype (Figure 9D). RA is involved in a variety of processes during early embryonic development. These include cell proliferation and differentiation as well as organogenesis [83]. Our results agreed with a previous study that demonstrated the ability of AuNPs to enhance the fate of the specification of ESCs to dopaminergic neurons by the involvement of the mammalian target of the rapamycin/p70S6K signaling pathway [84]. Recently, Han et al. [85] reported that silver NPs induce F9 cell differentiation in a dose-dependent manner. When undifferentiated human MSCs (hMSCs) were exposed to different concentrations of 10 and 80 nm AuNPs, no significant effect was evident on the proliferation of hMSCs [86]. Two prior studies reported the effect of AuNPs on adipogenic differentiation of murine MSCs and hMSCs [8,87]. Human ESCs were exposed to various 1.5, 4, and 14 nm diameter AuNPs and various assays were performed to assess the viability, pluripotency, neuronal differentiation, and DNA methylation of hESCs. The hESCs exposed to 1.5-nm diameter thiolate-capped AuNPs exhibited a loss of cohesiveness and detachment, suggesting ongoing cell death at concentrations as low as 0.1 μg/mL. Cells exposed to 1.5 nm AuNPs at 0.1 μg/mL did not form embryoid bodies, but rather disintegrated into single cells within 48 h. Cell death was also induced, whereas the other sized NPs were not toxic on the hESCs at concentrations up to 10 μg/mL during a 19-day neural differentiation period [88]. The collective data suggest that AuNPs can potentially induce differentiation in F9 cells without any alteration in cell viability and proliferation. However, the differentiation efficiency depends on the type of cell and size of the AuNPs.

### 2.9. Effect of AuNPs and RA on Expression of Differentiation and Stem Cell Markers in F9 Cells

**Figure 10:**
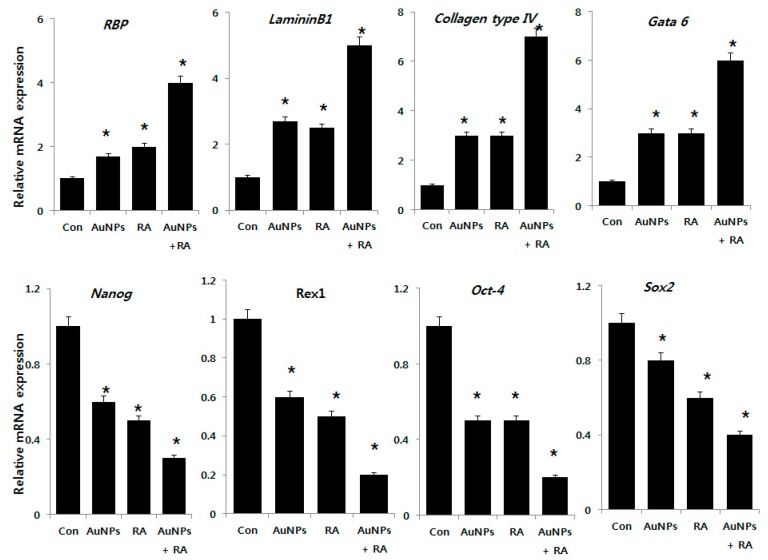
Analysis of expression of various differentiation and pluripotency stem cell markers. AuNPs-induced differentiation of F9 was determined after 24 h exposure to AuNPs (10 μM), RA (10 μM), and RA in the presence of AuNPs (both 10 μM). The expression pattern of the differentiation markers *retinoic acid binding protein (RBP)*, *laminin 1*, *collagen type IV*, and *Gata6* and stem cell pluripotency markers *Nanog*, *Rex1*, *Oct-4*, and *Sox-2* were analyzed in cells exposed to AuNPs (10 μM), RA (10 μM), and RA in the presence of AuNPs (both 10 μM). After 24 h treatment, the expression level was determined as fold-changes in reference to expression values of GAPDH. At least three independent experiments were performed for each sample. The treated groups showed statistically significant differences from the control group by the Student’s *t*-test (* *p*, 0.05).

To understand the mechanisms that regulate the balance between the proliferation and differentiation processes in teratocarcinoma stem cells, a comparative study of AuNPs and RA-induced differentiation of teratocarcinoma (EC) cells was conducted in the presence of AuNPs alone, RA alone, or RA in the presence of AuNPs. The cells were treated with AuNPs (10 μM) and RA (10 μM) and RA in the presence of AuNPs (both 10 μM) for 24 h and the expression level of the differentiation markers *retinoic acid binding protein (RBP)*, *laminin 1*, *collagen type IV*, and *Gata6* and the stem cell pluripotency markers *Nanog, Rex1*, *Oct-4*, and *Sox-2* were analyzed using RT-PCR. As shown in the top panel of Figure 10, quantification of mRNAs indicated significant differences in the expression levels of the differentiation markers compared to the control group. As we expected, AuNPs appeared to induce the expression of differentiation markers comparable with RA-treated cells. In addition, we analyzed the effect of AuNPs alone, RA alone, and both AuNPs and RA on the expression of the pluripotency markers. AuNPs or RA significantly reduced the expression of *Nanog*, *Rex1*, *Oct-4*, and *Sox-2* (Figure 10, bottom panel). The results suggest that F9 cells treated with AuNPs alone, RA alone, or RA in the presence of AuNPs significantly downregulated the expressions of the tested pluripotency genes. RA can induce the differentiation of EC and ES cells into primitive endoderm-like cells with the downregulation of pluripotency markers like Rex1 [89]. Woo et al. [90] developed an alternative approach to induce differentiation of hESCs, in which electrical stimulation was applied in the presence of fibronectin-coated AuNPs. The cells exhibited a loss of the expression of the Oct-4 stem cell marker and enhanced expression of the osteogenic markers collagen type I and Cbfa1. Recently, Han et al. [85] reported that F9 cells treated with lower concentrations of silver NPs displayed induced neuronal differentiation that was evident with the increased expression of various differentiation markers including *RBP*, *laminin B1*, and *collagen type IV* and the decreased expression of stem cell markers including *Nanog*, *Oct4*, and *Rex1*. Our results agreed with the previous studies. Another study demonstrated a concentration- and size-dependent effect of AuNPs on human ESCs, in which the cells were treated with two 4 and 14 nm AuNPs at 10 μg/mL. The expressions of *NCAM*, *NESTIN*, *BRACHYURY*, *PITX2*, *LEFTY*, *NODAL*, and *AFP* were not significantly altered. The authors concluded that the tested AuNPs did not markedly alter the in vitro differentiation potentials of hESCs [88]. Gordeeva and Khaydukov [91] explored the mechanisms of incomplete differentiation in a comparative study of RA-induced differentiation of mouse ESCs and teratocarcinoma (EC) cells. Higher expression of *Nanog*, *Mvh*, *Activin A*, and *BMP4* were evident in undifferentiated ESCs compared to EC cells.

The collective data indicates that AuNPs are a potential differentiation agent that could overcome RA-induced death of F9 cells.

## 3. Materials and Methods

### 3.1. Synthesis and Characterization of AuNPs

Synthesis and characterization of AuNPs was carried out as previously described [32]. AuNPs were synthesized by incubating 20 μM luteolin in 100 mL of water containing 1 mM HAuCl_4_ at 40 °C for 2 h. The color change from pale yellow to purple was due to the formation of AuNPs in the reaction mixture.

### 3.2. NO Measurement

NO measurement was performed as described previously [92,93]. F9 cells were treated with AuNPs (10 μM), RA (10 μM), RA in the presence of AuNPs (both 10 μM), or cisplatin (10 μM) for 24 h. The nitrite oxide levels in the medium were measured as an indicator of NO production based on the Griess reaction. Cell culture medium (75 μL) was mixed with an equal volume of Griess reagent and incubated at room temperature for 15 min. The absorbance at 540 nm was measured in a microplate reader. Fresh culture medium was used as the blank in all experiments.

### 3.3. Mitochondrial Transmembrane Potential (MTP) Assay

F9 cells were treated with AuNPs (10 μM), RA (10 μM), RA in the presence of AuNPs (both 10 μM), or cisplatin (10 μM) for 24 h. The change in MTP was determined using the cationic fluorescent dye JC-1 (Molecular Probes). Fluorescence of JC-1 aggregates and JC-1 monomers was measured at an excitation wavelength of 488 nm and an emission wavelength of 583 or 525 nm, respectively, using the aforementioned Gemini EM fluorescence microplate reader.

### 3.4. Measurement of ATP

The ATP level was measured according to the manufacturer’s instructions (Sigma-Aldrich Catalog Number MAK135, St. Louis, MO, USA) in F9 cells exposed to AuNPs (10 μM), RA (10 μM), or RA in the presence of AuNPs (both 10 μM) for 24 h.

### 3.5. Measurement of Anti-Oxidative Stress Markers

The anti-oxidative stress markers thioredoxin, GSH, GSSG, SOD, CAT, and GPx were assayed with reagents from various kits, according to each manufacturer’s instructions. Briefly, the cells were cultured in 75 cm^2^ culture flasks and exposed to AuNPs (10 μM), RA (10 μM), RA in the presence of AuNPs (both 10 μM), or cisplatin (10 μM) for 24 h. The cells were harvested in chilled PBS, by scraping and washing twice with 1 × PBS at 4 °C for 6 min at 1500 rpm. The cell pellet was sonicated at 15 W for 10 s (three cycles) to obtain the cell lysate. The resulting supernatant was stored at −70 °C until analyzed.

### 3.6. Reverse Transcription-Quantitative Polymerase Chain Reaction (RT-qPCR)

Total RNA was extracted from the cells treated with 10 μM of AuNPs, RA, and cisplatin for 24 h using the PicoPure RNA isolation kit (Arcturus Bioscience, Mountain View, CA, USA). Samples were prepared according to the manufacturer’s instructions. Real-time RT-qPCR was conducted using a Vill7 (Applied Biosystems, Foster City, CA, USA) and SYBR Green as the double-stranded DNA-specific fluorescent dye (Applied Biosystems, Foster City, CA, USA). Target gene expression levels were normalized to the expression of *glyceraldehyde-3-phosphate dehydrogenase* (*GAPDH)* expression, which was unaffected by treatment. The RT-PCR primer sets are shown in Table S1. Real-time RT-qPCR was performed independently in triplicate for each of the different samples. The data are presented as the mean values of gene expression measured in treated samples versus the control.

### 3.7. Statistical Analyses

Independent experiments were repeated at least three times. The data are presented as mean ± SD for all duplicates within an individual experiment. Data were analyzed by the Student’s *t*-test or multivariate analysis or one-way analysis of variance (ANOVA) and followed by the Tukey test for multiple comparisons to determine the differences between groups. Statistically significant differences are denoted by an asterisk. The analyses were performed using GraphPad Prism analysis software (GraphPad, La Jolla, CA, USA).

## 4. Conclusions

The controlled geometrical and optical properties of AuNPs are exploited in several applications including catalysis, electronics, photodynamic therapy, drug delivery, sensors, bio-imaging, and diagnosis. RA is a morphogen that plays important roles in cell growth, differentiation, organogenesis, and cancer treatment. Retinoids are a micronutrient necessary in the human diet to maintain several cellular functions. However, vitamin A can be toxic to the redox environment and mitochondrial functions. In the present study, we investigated whether AuNPs have protective actions against oxidative stress-induced damages by RA in teratocarcinoma stem cells. AuNPs were prepared using luteolin as a reducing and stabilizing agent. The synthesized particles were consistently spherical with an average and homogenous size of 18 nm. The viability of F9 cells treated with various concentrations of AuNPs was unaffected. RA was toxic, it diminished cell viability and inhibited cell proliferation in a dose-dependent manner. Past studies have demonstrated that the toxic or beneficial effects of AuNPs on cells depend on their shape, surface charge, functionalization, and biological viability. The major factor for RA-induced toxicity is the increased levels of LDH, ROS, MDA, and NO; the loss of MMP; and the reduced level of ATP. RA increased the level of pro-apoptotic gene expression and decreased the expression level of anti-apoptotic genes and concurrently decreased the expression level of anti-oxidant genes. Interestingly, AuNPs not only ameliorated the oxidative stress but also induced differentiation in F9 cells by increasing the expression of differentiation markers including *RBP*, *laminin 1*, *collagen type IV*, and *Gata 6* and decreasing the expression of pluripotent stem cell markers *Nanog*, *Rex1*, *Oct-4*, and *Sox-2*. AuNPs significantly inhibited RA-induced toxicity and similarly exerted a positive effect on the differentiation of F9 cells. Considering both the ability of AuNPs to reduce the level of oxidative stress and the important role of differentiation, the overall data we present implicate AuNPs as a suitable therapeutic agent for oxidative stress-related diseases including atherosclerosis, cancer, diabetics, rheumatoid arthritis, and neurodegenerative diseases. Further studies are required to explore the underlying mechanism of AuNPs as an anti-oxidative and differentiation agent.

## Supplementary Materials

The following are available online at http://www.mdpi.com/2079-4991/8/6/396/s1; Table S1: Supplementary materials and methods.
