# Supplementary material for: Biocompatible Gold Nanoparticles Ameliorate Retinoic Acid-Induced Cell Death and Induce Differentiation in F9 Teratocarcinoma Stem Cells
Source: Nanomaterials (Basel). 2018 Jun 1;8(6):396. doi: 10.3390/nano8060396 (PMC6027053; doi:10.3390/nano8060396)
Supplement: Supplementary file 1 [file nanomaterials-08-00396-s001.pdf]

Supplementary

Materials

Dulbecco's modified Eagle's medium (DMEM), penicillin-streptomycin solution, and trypsin-EDTA solution were purchased from Gibco (Grand Island, NY, USA). Fetal bovine serum (FBS), in vitro cell proliferation kit, and cell-counting kit-8 (CCK8) were obtained from DojindoLaboratories (Kumamoto, Japan). In Vitro Toxicology Assay Kit (TOX7), 2′,7′-dichlorodihydrofluorescein diacetate (H2-DCFDA), and cationic fluorescent indicator (JC-1) were obtained from Molecular Probes (Eugene, OR, USA). Luteolin, retinoic acid, gold (III) chloride trihydrate, and in vitro toxicology assay kit were purchased from Sigma-Aldrich (St. Louis, MO, USA). All other chemicals were purchased from Sigma-Aldrich unless otherwise stated.

Cell culture and treatment

F9 mouse EC cells were purchased from the Korean Cell Line Bank (KCLB) and were maintained in DMEM supplemented with 10% FBS and 1% antibiotic-antimycotic solution. Cells were grown to confluence at 37°C in 5% CO_2. Experiments were performed in 96-, 24-, and 12-well plates and 100-mm cell culture dishes, as the experiment demanded. Cells were treated with AuNPs (10 μM), RA (10 μM), RA in the presence of AuNPs (both 10 μM), or cisplatin (10 μM).

Cell viability

Cell viability was measured using a Cell Counting Kit-8 (CCK-8; CK04-01, Dojindo Laboratories). Briefly, F9 cells were plated in 96-well flat-bottom culture plates containing various concentrations of AuNPs or RA, or RA in the presence of AuNPs (both 10 μM). After a 24 h culture at 37°C and 5% CO_2 in a humidified incubator, CCK-8 solution (10 μL) was added to each well and the plate was incubated for another 2 h at 37°C. The absorbance was measured at 450 nm using a Multiskan FC microplate reader (Thermo Fisher Scientific Inc., Waltham, MA, USA).

BrdU cell proliferation assay

Cell proliferation was determined according to the manufacturer's instructions (Roche, Basel, Switzerland). Cells were incubated with various concentrations of AuNPs or RA, or RA in the presence of AuNPs (both 10 μM) for 24 h. BrdU labeling solution was added to the culture medium 2 h before the end of the incubation. Cells were fixed and the level of incorporated BrdU was determined using the Cell Proliferation ELISA BrdU assay kit (Roche) following the manufacturer's instructions. Proliferation activity of the untreated cells at the time point of 0 h was considered as 100%. Differentiation experiments were carried out in the culture medium supplemented with 1% FBS with AuNPs (10 μM) or RA (10 μM) or RA in the presence of AuNPs (both 10 μM) for 24 h.

Membrane integrity

The membrane integrity of F9 cells was evaluated using the LDH Cytotoxicity Detection Kit. Briefly, cells were exposed to various concentrations of AuNPs for 24 h. Subsequently, 100 μL of cell-free supernatant from each well was transferred in triplicate into the wells of a 96-well plate and 100 μL of the LDH reaction mixture was added to each well. After 3 h incubation under standard conditions, the optical density of the final solution was determined at a wavelength of 490 nm using a microplate reader.

Determination of intracellular ROS

F9 cells were treated with AuNPs (10 μM), RA (10 μM), RA in the presence of AuNPs (both 10 μM), or cisplatin (10 μM) for 24 h. ROS was measured according to a previous method based on the intracellular peroxide-dependent oxidation of 2′,7′-dichlorodihydrofluorescein diacetate (DCFH-DA, Molecular Probes) to form the fluorescent compound 2′,7′-dichlorofluorescein (DCF) [92]. Cells were seeded in wells of 24-well plates at a density of 5×10^4 cells per well and cultured for 24 h. After washing twice with PBS, fresh medium containing AuNPs (10 μM), RA (10 μM), RA in the presence of AuNPs (both 10 μM), or cisplatin (10 μM) was added, and the cells were incubated for 3 h. For the control, 20 μM of DCFH-DA was added to the cells and incubated for a further 30 min at 37°C. The cells were rinsed with PBS, 2 mL of PBS was added to each well, and the fluorescence intensity was determined using a Gemini EM fluorescence microplate reader (Molecular Devices, Sunnyvale, CA, USA) at excitation and emission wavelengths of 485 nm and 530 nm, respectively. DCFH-DA (20 μM) was then added and the cells were incubated for 30 min at 37°C before measuring the changes in DCF fluorescence.

Measurement of MDA

F9 cells were treated with AuNPs (10 μM), RA (10 μM), RA in the presence of AuNPs (both 10 μM), or cisplatin (10 μM) for 24 h and MDA was measured as described in the manufacturer’s instructions (Sigma-Aldrich; Catalog Number MAK085). LPO was determined by the reaction of MDA with thiobarbituric acid (TBA) to form a colorimetric (532 nm)/fluorometric (excitation and emission wavelength of 532 nm and 553 nm, respectively) product, whose quantity was proportional to the MDA present.

Suppl. Table 1. List of primers used in this study

Name of Gene	Primer sequences
Nanog	F:CAGGCCTGGATGAAGAAGAG
	R:ACTGGTAGAAGAATCAGGGCT
Rex1	F:AAGCAGGATCGCCTCACTGT
	R:GCTTCCAGAACCTGGCGAGA
P53	F:AGAGACCGTACAGAAGA
	R:CTGTAGCATGGGATCCTTT
P21	F:GTTGCTGTCCGGACTACCG
	R:AAAAACAATGCCACCACTCC
Caspase-3	F:AGGGGTCATTTATGGGACA
	R:TACACGGGATCTGTTTCTTTG
Caspase-9	F:GTCACGGCTTTGATGGAGAT
	R:CAGGCCTGGATGAAGAAGAG
Bax	F:CGAGCTGATCAGAACCATCA
	R:GAAAAATGCCTTTCCCCTTC
Oct4	F:CTCCCTACAGCAGATCACTCACA
	R:AACCATACTCGAACCACATCCT
Bcl-2	F:TAAGCTGTCACAGAGGGGCT
	R:TGAAGAGTTCCTCCACCACC
RBP	F:GACAAGGCTCGTTTCTCTGG
	R:AAAGGAGGCTACACCCCAGT
Laminin B1	F:ACAACACCAAAGGCCTGAAC
	R:TGCCAGTAGCCAGGAAGACT
Collagen type IV	F:AAAGGGAGAAAGAGGCTTGC
	R:CCTTTGTACCGTTGCATCCT
Bak	F:CTC AGA GTT CCA GAC CAT GTT G
	R:CAT GCT GGT AGA CGT GTA GGG
Gata 6	F:TTCTAACTCAGATGATTGCAGC
	R:GCTGCACAAAAGCAGACACG
Sox2	F:GCCTGGGCGCCGAGTGGA
	R:GGGCGAGCCGTTCATGTAGGTCTG
GAPDH	F:AGGTCGGTGTGAACGGATTTG
	R:TGTAGACCATGTAGTTGAGGTCA
